# Abstract of the Proceedings of the Buffalo Medical Association

**Published:** 1858-09

**Authors:** Austin Flint


ART. III.—*Abstract of Proceedings of the Buffalo Medical Association.*

TUESDAY EVENING, Aug. 3, 1858.

The Association met.

Present—The President, Dr. Wyckoff, in the chair; Drs. Wilcox, White, Flint, Nichell, Newman, Strong, Gay, Hutchins, Nott, Miner, Ring, Lemon, Flint, Jr., and Drs. Ham, of Williamsville, and Rogers, honorary members.

The reading of the minutes was deferred until the next meeting.

On motion, the following gentlemen, whose names were presented for membership at the last meeting, were declared members of the association, *i. e.*, Drs. Butler, Lockwood, Jansen, Stevens and Richards.

Dr. HAM, of Williamsville, then read the following report on "Hæmorrhage from the Ear as a Diagnostic Sign of Fracture of the Base of the Skull."

Much having been said, of late, by authors and in the journals, upon hæmorrhage from the ears as diagnostic of fractures of the base of the skull,

I will in short give my views and experience upon the question. As a basis upon which to found the present observations, I will give a case and illustrate the points as they come before us.

On the 16th day of July passed, N. I., æt. 24, farmer, of good general health, robust, and of good habits, received a violent kick from a powerful young horse, upon the left temporal region. The injury was inflicted at 6 o'clock in the morning.

He was in a pasture, some half mile from home, when the blow was received. He was carried to his residence upon a litter, in a helpless and senseless condition. I saw the patient with D. W. Horshey, M. D., my business partner, at 10.30, A. M. He could then speak; could give no connected account of accident; was much confused; staring look; surface pale and cold, the extremities very much so. Blood had discharged very freely from the external wound, from the left ear and from the throat. The soft parts were much bruised and greatly tumefied. A deep cut was found in front of the ear, into which a probe could be passed to the bone in front of the external meatus, but could not be passed into the meatus; hence our opinion was that the hæmorrhage was from the deep seated parts of the internal ear, and this opinion was confirmed by the free discharge of blood through the eustachian tube into the throat. No fracture or depression was to be found. A paralysis of the left side of the face showed itself.

The patient vomited at intervals, which was looked upon as a favorable symptom, showing that reäction had already taken place. I chose to neither bleed or to give strong stimulants, lest the powers of life should be still further depressed by the former, or my patient strangled by the use of the latter. Recommended mild stimulants and gentle frictions, till reäction should be fully restored.

Saw the patient at 6 o'clock, P. M. Breathing better; pulse more full; extremities warmer; surface less pale; reäction considerably restored; hæmorrhage from the ear still less.

July 17, 9 o'clock, A.M. Reäction fully restored; pulse full. Gave cath.

℞ Cal., 10 gr.
Jalap, 10 gr.

Also,

℞ Tart. ant., 4 gr.

Spt. nit. dul., 1 ʒ.

Syrup ipecac, 1 ʒ.

Water, 1 ℥. M.

Dose — teaspoonful every two hours.

Directed cold to the head; warmth to the feet; enjoined perfect quiet.

During the night of the 17th, there was a free discharge of blood from the right ear.

July 18th. Cath. had operated well.

Treatment continued.

More pain in the head; intolerance to light. Applied ice to the head.

At 4.15, P. M., a small quantity of coagula was vomited, which was probably taken into the stomach in the early part of the injury. Patient moans and is quite restless; much pain in the head; pulse full and bounding; paralysis of the face much increased.

Bled 16 oz.

At this visit there was discovered great difficulty in closing the left eye. From this time forward the eye constantly rolled upward and inward.

July 19th. Patient had a good night, and in all respects appears considerably improved. There was no discharge of blood, from this time, either from the ear, nose or the throat. Hope and courage for a favorable termination on the increase.

Treatment continued.

July 20th. Patient had a restless night. Before my daily visit, my friend W. Van Pelt, M. D., was going by, was called in and very properly bled again to the amount of 16 oz., which had the effect of relieving the patient of present urgent symptoms. At my afternoon visit, found subsultus and great restlessness. Put the patient upon fluid ext. hyoscyamus, and caused to give alterative doses of hydr. sub. mur., and applied a large blister to the left arm.

July 21st. Same.

Treatment continued.

July 22d. All the symptoms aggravated; the case from this time became urgent and alarming; the mouth draws far to the right side; the tongue paralyzed, and protruded with great difficulty, is thrust far to the right side; the uvula drawn much to the right, and insensible to the touch. Active inflammation is present. The patient failed; became typhoid; and died at 11 o'clock, Wednesday P. M., the 28th ult., and the thirteenth day after the injury. No post-mortem could be had.

Here are all the material facts in the case. Here is the blow; the insensibility; the bleeding from both ears and from the mouth; paralysis of the face, the eye, the tongue, and the uvula. No external fracture.

The question now comes up,—Was there fracture of the petrous portion of the temporal bone, or elsewhere?

No greater truth in surgery is, now better understood and established, than that the bad symptoms very frequently accompanying a broken skull, are not produced by the breach made in the bone, nor do they indicate such breach to have been made.

Sir Astley Cooper, (*Lectures, &c., page* 289,) remarks, "The danger of fractures of the skull depends upon their being united with concussion or extravasation; there is also a remote danger of inflammation." This was also the opinion of Pott, who observes, "The sickness, vomiting, and loss of sense and motion can only be the consequence of an affection of the brain as a common sensorium. They may be produced by having been violently shaken, by a derangement of its medullary structure, or by unnatural pressure made by a fluid extravasated on its surface, or within its ventricles; but never can be caused by the mere division of the bone, which division in a simple fracture, can neither press on nor derange the structure of the parts within the cranium."

Should we assume the hypothesis that the base was fractured in this case, the query then arises, how was it done? You will observe, *there was no external fracture or depression.*

M. Aran, (*Med. Gaz. for* 1845, *p.* 364,) combats, *in toto*, the ancient and still prevailing opinion of fractures of the cranium, by what are called *contre coups;* where the base, for example, is fractured by a blow on an opposite or distant part.

M. Aran classes all these fractures by *contre-coup* under two heads, viz., 1st. Those that are *independent*, *i. e.*, where the part struck as well as the distant one, are both fractured; 2d. Where the fracture is produced by *irradiation* or prolongation from the place struck, and fractured to the base of the cranium.

M. Aran has made a great number of experiments on the heads of dead subjects, striking them with hammers, or precipitating them from certain heights *head foremost.* These are his principle conclusions. He has never known a fracture at the base without one at the point struck, also. He has seen no fractures of the base by *contre-coup.*

Fractures of the base of the cranium usually arrive there by irradiation and by the shortest curve. Fractures by irradiation constitute ninety-nine out of every hundred at the base.

Cormack's London and Edinburgh Journal, June, 1855, page 461, gives a case which came under the treatment of M. Blandin, of a man, aged thirty, who, in a fall, fractured the petrous portion of the temporal bone, causing hæmorrhage from the left ear and from the mouth, with slight defect in hearing; paralysis of that side of the face, with paralysis of the uvula to such an extent that it was drawn to the right side of the base of the tongue, by its muscles of that side now having no antagonists. This case, in all its main features, is in point with that of my own. M. Monteau conceives that this case has apparently established an important, but hitherto obscure point in anatomy, and thus furnished a new diagnostic mark in pathology in such injuries.

It is known that the uvula receives its nerves from the spheno-palatine ganglion by three filaments, which go from this ganglion to the uvula. But the ganglion itself also receives a filament from the intra-cranial portion of the facial nerve, as has been clearly shown by M. Blendin. Our author says: "This, however, has been *erroneously* supposed to be a filament *sent from* the ganglion to the vidian nerve; whereas, it is now found to be a distributor of nervous influence from the vidian nerve to the uvula. From whence we have the key to the condition of these parts in the fractures in question, and the explanation why lesions of the intra-cranial trunk of the facial nerve must produce paralysis in the filament which goes to the spheno-palatine ganglion, and afterwards leaves it as the motor nerve of the uvula." From the foregoing anatomical fact, M. Blendin argues that (*see Velpeau's Operative Surgery, by Mott, vol.* 2, *p.* 934,) the uvula is only paralyzed when the cause of the paralysis of the face is within the cranium, close by the petrous portion of the temporal bone. Therefore, we have this valuable diagnostic: when there is paralysis of the face alone, without accompanying paralysis of the uvula, we may affirm that the lesion is external, or in the peripheral branches of the nerve. He considers hæmorrhage from the ear, the nose and the mouth, in such cases, as unequivocal signs of fracture of the petrous bone. He thinks the blood emanating from the internal ear, where it is extravasated and escapes anteriorly by the external ear, and posteriorly by the eustachian tube, through which it finds its way into the throat. M. Blandin has verified these conclusions by numerous dissections.

On the other hand, Dr. Lawrie has recorded *twenty-two* cases of cerebral concussion, (Cormack, page 462, also page 673, for 1843,) at the Royal Infirmary, at Glasgow, in which there was hæmorrhage both from the ear and the mouth, and yet *twenty* of those cases recovered; and in *one* only of the *two* fatal cases was there fracture of the base of the skull to be found on dissection.

Dr. Cormack speaks of other similar cases which recovered, which places in great doubt the opinion that hæmorrhage from the ears and the throat is conclusive evidence of fracture of the petrous or any other bone.

M. Gordy, (*Lond. and Edin. Journ.* for 1845, p. 463,) is stated to concur also in the opinion that the hæmorrhages in question do not always indicate fracture.

Again, (same Journal, 1845, p. 634,) we have three illustrative cases, two by Gordy himself, and one by Velpeau, all of which recovered.

The editors of the Paris Journal, in which all these and many more cases are given (*Annales de Therap.*) conclude from all the above facts, that in the present state of science, hæmorrhage from the ear under the circumstances, even though the hæmorrhage may by accompanied by paralysis of the face and the uvula, does not permit us to pronounce that a fracture exists.

Prof. WHITE related a case which had occurred in this city, and which had excited a great deal of interest, from its peculiarity, and the fact that the patient instituted a suit against the person who had injured him. As he was passing along the street, a brick, falling from the top of a four story building, struck him on the top of the head. On examination, no fracture was discovered in that situation, and the skin even was not broken; but there existed a fracture of the skull on both sides of the head. He was taken up senseless, and had hæmorrhage from the throat. The man recovered from the injury, and subsequently died of cholera. The case is interesting as illustrating the fracture by *contre-coup.*

Dr. NOTT remarked that he had made a post-mortem examination in a case where there were external bruises of the scalp, but no external fracture of the skull. On opening the head, however, a fracture of the inner table of the petrous portion of the temporal bone was discovered. Death resulted by hæmorrhage from the meningial artery. In this case there was no external fracture or injury to the bone.

Dr. MINER then read the following report of a case of Traumatic Tetanus:

March 2d, 1858. Mr. Harris, of this city, aged 45 years; robust and healthy; received an injury of the hand by being caught in a mill used in grinding charred bones; by this means the integument was mostly removed from the palmar aspect of all the fingers to the hand; the muscles were much lacerated, and the flexor tendons of the third finger were completely removed, leaving the bones bare, yet producing no fracture.

In consultation with Drs. Hamilton and Newman, after placing the patient under the influence of chloroform, removing the fragments of torn flesh and tendon, ligaturing some small vessels which kept up continual hæmorrhage, and cleaning the wound of the coal which had been ground into it, giving the appearance of a gun-shot wound, desiring to save, if possible, the hand entire, and also wishing to give the patient opportunity of choice between stiff and imperfect fingers and no fingers at all, it was decided to place the hand in a poultice, with the view that subsequent amputation be made, should the injury be found so great, or the supply of blood so much obstructed, as to render repair impossible.

March 18th. Sixteen days after injury. Up to this date, nothing of importance had occurred which would not have been anticipated. The pain, though severe, was not greater than would be expected in such an injury; rest had been obtained by use of morphine; hand had been in poultice most of the time; granulations healthy in appearance; the wound in all respects progressing favorably. The strength had been supported by sustaining diet, quinine, and brandy.

When about to leave, after dressing the hand, Mr. H. remarked carelessly, "Dr., I cannot open my mouth so *quick* as common." *This* was all he complained of, after careful inquiry, and accounted for this, even, in having taken cold while sitting up the day previous, to himself quite satisfactorily, but to me quite the reverse.

March 22d. Dr. Hamilton in consultation. For the past four days, tetanic symptoms have been gradually increasing in severity; jaws, open a little with great effort, are for the most part firmly closed; muscles of the whole system somewhat rigid, and lost to the ready control of the will; pain in the epigastric region extending through to the spine, at times very severe; skin cool and bathed in perspiration; pulse 120 per minute, and quite feeble; mind clear when fully awake, with much talking when asleep; hand still looking very well; healthy granulations have nearly filled the deepest excavations, the slighter injuries completely healed; bowels readily moved by common physic or injections, with offensive discharges.

March 28th. The disease has now reached its acme. Pulse 130 per minute, very feeble; skin bathed in profuse perspiration, with cold hands and feet; urine scanty, and passed with great effort and pain; tongue moist as far as may be seen; mouth filled with viscid mucus; epigastric pain very severe, with nausea and vomiting, causing great distress, on account of the impossibility of discharging the contents of the stomach through the mouth; patient greatly disturbed by slightest causes; still no delirium, but much talking when asleep; electric-like shocks of spasmodic action convulse the whole system upon the least effort to move, or when moved, and at intervals of from fifteen to thirty minutes when perfectly unmolested, sometimes drawing the patient backward so as to rest upon back of head and heels. This condition of spasm continues but a moment, yet is complained of as producing great pain. The third finger, which for a few days has been enlarged and inflamed, now discharges unhealthy pus by two fistulous openings near both the first and second joint; by probing, I discover caries of the bone, or bone denuded of periosteum.

April 20th. For the last twenty days there has been gradual improvement. The muscles of the trunk and abdomen more relaxed than those of the legs. The legs are kept firmly extended and held by attendan's, lest the heels be drawn against the nates, as is often the case when great care is not used to prevent it. Pulse reduced in frequency and increased in force; skin more natural, with extremities warm; expression of countenance cheerful, yet very characteristic of the disease; desire for food, which has never been wholly absent, is increased: it is now sometimes placed in the mouth, though mostly taken through a tube.

The opening at the first joint of the finger has become so large, the bones so separated, from attachments, and the system of the patient so much improved, that with strong forceps I removed the two last phalanges through the opening, an operation I have had a desire to make for several days, delayed by the fears of suffering in my patient, and my own fear that the spasms be renewed.

April 28th. From the time of removing the diseased bones, there has been the most improvement. The finger is assuming its natural size, the openings are closed, and the patient is proposing a journey to the country to-morrow.

I have reported the condition of this patient in a few representative days, desiring to show the appearance, progress, and termination of a case of disease, interesting from the usual obscurity of its causes, the great infrequency of its appearance, at least in our own climate, and from its almost unequaled fatality, rather than for the purpose of showing by what new or wonderful treatment my patient has recovered from this formidable disease; perhaps, however, I should say, that tonics, stimulants, and sustaining diet, with anodynes, constituted the main reliance. During the severity of the disease, two grains acetate morphia, every four hours in injection, *seemed* to be attended by the happiest effects Chloroform was only administered at the first dressing, on account of the absence of continuous violent spasm, and the low depressed state of the system.

There is no disease in which prophylactic and curative measures have been so extensively used as tetanus; and none in which it is more difficult to determine what influence they have exerted. When our ignorance of the pathology of tetanus is taken into consideration, we need not be surprised that the treatment in many cases is strictly empirical; neither need we wonder that the most opposite remedies have been occasionally used with apparent success, and each in turn been rejected as unworthy of confidence.

The exciting cause in this case is very manifest; constitutional pecularity, or other predisposing influences may have acted upon the nervous system, yet nothing was visible in the least indicating tetanus or violent constitutional disturbance, until about the time when the finger commenced swelling and giving indications of diseased structure within. When the inflammatory action had reached its height, and discharge was fully established, the violence of the spasms commenced gradually to subside; and when all the diseased bone was removed, the rapid subsidence of all diseased action indicated most unmistakably the cause of the disturbance.

This is worthy of note, since most cases of tetanus arise from inappreciable causes, or follow injuries or operations, when dissection reveals no uncommon source of irritation. Had death taken place very early, it might have been so even in this case.

Four months after injury, I have examined the finger, and much to my surprise find the second bone of the finger has been reproduced, the first only is now absent. The new one is about two-thirds as long as the one removed.

Prof. White reported an interesting case of tetanus which had occurred in his practice. The following is an account of the case taken from the note book of Dr. Flint, who saw the patient with Dr. White:

August 21, 1857. Visited yesterday with Dr. White, a child on Michigan street, aged 7 years. About a fortnight ago, he was attacked with his present affection, and the disease has remained ever since about stationary. About every half hour, he is seized with opisthotonic spasms; the body is curved backward; the penis is erected; muscles of abdomen rigid; the arms rigid with the thumbs pronated; and the jaws firmly closed. No embarrassment of respiration apparent. The paroxysms last two or three minutes and gradually pass off. They recur spontaneously every few minutes; oftener during the night than during the day. They can always be brought on by raising the body. When the opisthotonos occurs, the entire body can be lifted by raising one lower extremity. A certain amount of trismus is constant; he cannot open the mouth so as to protrude the tongue or to take food freely. The face grimaces with the angles of the mouth depressed, presenting an aspect of intense distress. He appears to suffer severe pain during the paroxysms. The mind is clear. He obtains but little sleep. Pulse not accelerated. The surface of the body presents numerous red papules. The frequency and severity of the paroxysms have neither increased nor diminished in a notable degree, but continue about the same.

The affection cannot be distinctly traced to any cause. He has not received any wound, and before this, seemed well. The mother is inclined to attribute it to a fright—a neighbor telling him shortly before that he would kill him, and showing a butcher knife. The boy, however, says he was not much frightened. The mother is an ignorant Irish woman, and it is not easy to obtain a clear account of the manner in which the disease was developed.

The remedies given by Dr. White were quinia, valerianate of zinc, and morphia. It was proposed to-day to try the effect of chloroform.

August 25th. The chloroform was given for two or three hours with the effect of tranquilizing the patient; relaxing partially the trismus and prolonging the intervals between the paroxysms. The mother was to have sent word the next morning how the child was: she failed to do so, and Dr. White did not see the patient or hear from him since the evening of the 21st.

September 8th, 1857. The patient has nearly recovered. He still occasionally has slight opisthotonic spasms when excited, but is up and about, and evidently convalescing. Since the trial of chloroform on the 21st ult., no treatment has been employed. The mother has contented herself with simply giving the child nutritious diet. No physician had seen it after the 21st ult., until Dr. Flint, Jr. called a few days since to ascertain the result.

Dr. Newman had seen the case reported to the association by Dr. Miner. He saw a fatal case of the same disease four years ago. In this instance, death took place instantaneously during a spasm. The patient was being raised up for the purpose of taking some medicine, and just as he was about to swallow, he had a spasm and died immediately. The disease had been developed for forty-eight hours. When he first saw the patient, Dr. Newman thought it was a case of mania potu, as he had been drinking hard, and the symptoms resembled that disease. He accordingly gave opiates and stimulants. On the next morning he found difficulty of swallowing and pain at the pit of the stomach. The patient had run a nail in the ball of the great toe some weeks before, but the wound had healed kindly.

Prof. Rochester had seen two cases, one of which was lost in the manner described by Dr. Newman. This case occurred in a middle-aged man, who was suffering from a cancer in the groin. The spasms were exceedingly severe. In an effort to swallow a dose of laudanum, he died instantaneously. The other case recovered. The patient received a severe injury between the thumb and forefinger. Tetanus was developed in two weeks. The cicatrix was laid open, and the patient treated with opium and brandy. The symptoms began to yield when free suppuration was established.

Dr. Wyckoff had seen a case which proved fatal. The treatment consisted of tonics, stimulants, etc. Great relief was experienced from smoking tobacco. He would smoke a pipe for hours, having it refilled and relighted without removing it from the mouth. The disease was attributed to a slight injury to the foot. The patient died after two or three weeks' sickness.

Prof. Flint moved that Dr. Miner be appointed a committee to report on the cases which had occurred in this city. Seconded and carried.

Prof. White then reported an interesting case of obstetrics. Mrs. ——, a German woman, æt 45 years. In her thirteenth pregnancy. Dr. White in consultation with Dr. Devening. The patient was very œdematous, with the legs very much swollen. The abdomen was excessively large and pendulous, hanging down some distance over the thighs. The uterus could be felt through the fat and effusion, hanging over the pelvis. She had expected her labor some weeks since, and had had pains for thirty or forty hours. Dr. Devening had made an examination but was unable to reach the os. She suffered greatly from pains, but the uterine tumor did not descend into the pelvis. On making an examination, Dr. W. was unable to reach the os without introducing his entire hand into the vagina, when he found it high up and pointed backward over the promentory of the sacrum. The os was dilated fully, and the membranes were protruding. Dr. Devening had applied a bandage so as to support the pendulous belly, and had placed the woman upon her back, as is recommended in such cases; still the uterus and its contents were so anteverted as to direct its axis upward and backward.

The membranes were now ruptured and the waters were copiously discharged. Dr. White then pulled forcibly upon the anterior lip of the os with his right hand, sitting between the woman's legs, her feet being supported by chairs at the side of the bed; at the same time he depressed her head, and Dr. Devening with both hands, assisted by the left hand of Dr. White, elevated the fundus. The cord descended into the vagina and Dr. W. introducing his hand into the uterus, passing by the head, which presented, seized the feet and delivered. The child was a female, medium size, and partially asphyxiated, but was revived by alternate dashes of hot and cold water.

Upon examining the abdomen, it was found still large and pendulous; and upon carrying the finger into the os, another bag of waters was discovered, which was ruptured and the arm of the second child descended into the vagina. Upon carrying the hand up to search for the feet, Dr. W. was obliged to pass it anteriorly, over the pelvic brim, and down into the sac in front of the pelvis; the arm being in the vagina. The fundus was again elevated in the same forcible manner, and with great difficulty the feet were brought into the vagina, and the child (female) delivered in a partially asphyxiated condition. It was soon restored, however, by dashes of hot and cold water.

The placenta was then delivered, and the patient placed in bed, and ergot given to procure uterine contraction. Both children together, weighed seventeen and one-half pounds.

Obliguity of the uterus is quite common, and is usually corrected merely by position. The case above reported was one of most extraordinary anterior flexion, and one which could never have been remedied by bandages and the supine position. The uterine tumor occupied a sort of a pouch over the pubes, and extended below the genital fissure and upon the thighs, with the fundus directed downward, and the os upward and backward, so that the contraction directed the bag of waters against the back, above the sacro-vertebral angle. In this case, it would have been impossible for delivery to have taken place, with this relation of the parts. The cause was probably the number of children which the woman had borne, the great weight of the tumor, and relaxation of the abdominal muscles.

Prof. WHITE also presented a case of vascular tumor of the female urethra. Rigby asserts that it is most common in young females, but Dr. White has seen two cases in persons over 60 years of age. The case which he presented was a female, æt. 54 years, a patient of Dr. Wilcox. She had a small vascular tumor, about the size of an apple seed, situated at the orifice of the urethra. It is of a bright red color, and exceedingly sensitive; every act of micturition being accompanied with excessive pain. Occasionally a drop or two of blood escapes with the urine. The patient was exceedingly sensitive and averse to an examination, but was at last forced to consent. Dr. Wilcox made a vaginal examination and discovered nothing; but on withdrawing his finger, it came in contact with this tumor, the existence of which he had not before suspected, causing intense suffering. She had complained of pain with micturition, accompanied by a thrilling sensation, and had used a syringe and a wash, but with no good effect. Dr. Wilcox then insisted upon an ocular examination, but it was two or three weeks after the vaginal examination, before it was permitted. He then discovered the tumor which has been described. Dr. White was called in, who administered chloroform, and removed the tumor by torsion. The patient was seen a month after and reported herself cured. Dr. White remarked that these tumors were not very common; the effects are entirely local, but they produced a great deal of distress. They can be cured by removal by torsion or caustics. Dr. West recommends the actual cautery, which is probably the most efficient means of treatment.

Dr. Ring stated that during a visit in the country, he had visited a patient, with several practitioners, in that neighborhood, that was of much interest to them. The patient was a gentleman nearly sixty years of age, of sanguine-bilious temperament, and above medium height and weight. The prominent symptom was *dyspnœa,* which was much increased by bodily or mental exercise. Physical exploration showed dullness on percussion over the entire right lung, where bronchial respiration was present. That side of the chest was somewhat flattened, and the intercostal spaces depressed, and not much movement during respiration. There was no effusion in the cellular tissue, or abdomen, and he was able to lie in the horizontal position without inconvenience. Heart and pulse natural so far as we could discover. In fact while the patient was so troubled with dyspnœa as to disqualify him for business, and to put him in dread of sudden death, and had been confined to his room for over six months, his appearance was nearly that of health, except that he was somewhat emaciated. On the same side, commencing about one and a half inches from the sternum, and over the fifth, sixth, and seventh ribs, is a fleshy, lobulated tumor, extending backwards and downwards on the ribs for three or four inches. This tumor first made its appearance about twelve years ago, after an attack of sickness, and since that time, the patient had difficulty of breathing after over exertion. In the absence of any *marked* symptoms of aneurism, of disease of the heart, effusion in the chest, or inflammatory hepatization of the lung, it was suggested that the solidification of the lung and consequent dyspnœa, was probably caused by the growth of the tumor internally. He inquired if any gentleman of the association present had ever had a case of mediastimal tumor under observation.

Dr. Miner had seen a case of fibrous tumor of the mediastinum, in a child. It enlarged so as to extend up to the neck, and the patient died by suffocation. A post-mortem examination was made, and the tumor was discovered.

Dr. Miner mentioned a case of the birth of a child without pain. Mrs. F. A. Strong, a robust and healthy woman, called upon him to attend her on July 12th, at 5, A. M; the waters having been discharged, and a little uneasiness felt in the back. The os uteri was found well dilated, but there were no labor pains. Dr. M. then left the patient till 12 o'clock, and as there were no pains then, gave Thayer's fluid extract of ergot in full doses, without effect. He saw the patient at 6, P. M., and found the child born and the placenta just discharged. In trying to turn in bed, the mother found that the contents of the uterus had passed from her with absolutely no pain. There was no hæmorrhage; the uterus contracted in the usual manner, and the recovery was complete. She had been delivered two years since with the forceps, by Prof. White, of a child which soon died.

Dr. Miner had known of one case like the preceding. He had attended a lady twice in an abortion at six months. The first time there was almost fatal hæmorrhage before he saw the patient. In both instances he removed the fœtus with the hand, and in neither case was there any pain.

Prof. White recollected the patient referred to by Dr. Miner. He had applied the forceps on account of atonicity of the uterus.

Prof. Flint thought that some allowance should be made in regard to such statements from patients. He had had two cases where the women said that there was no pain.

Prof. White presented another interesting case. Mrs. W., æt. 26, good constitution; in the sixth month of her third pregnancy. In her second confinement in June of last year, she had severe puerperal fever, from which the convalescence was gradual. This was succeeded by mental depression, constipation, headache, &c., but last month she was better than usual. On the 26th of May, she had breakfasted as usual, and was ironing, when she became suddenly sick and faint. She immediately lay down upon a lounge, vomited and complained of severe pain in the head. Dr. White saw her at 9½, a half hour after the attack. She was then semi-conscious; countenance flushed; pulse slow and labored; pupils alternately dilated and contracted. He then bled her, with the effect of increasing the frequency of the pulse; applied cold to the head and mustard to the feet. At 11, A. M., she was in profound coma and had not spoken since 10. Treatment continued, with one drop of croton oil every hour until operation. At 12½, Dr. Rochester was called in consultation. Pulse unsteady; pupils fixed; breathing stertorous, and capillary congestion of the surface. Continued the treatment and applied a mustard cataplasm to the neck. The patient died at 1½ o'clock, four and a-half hours after the first attack.

Post-mortem examination twenty-two hours after death. Examination confined to the head; vessels of scalp much congested with venous blood. Membranes also congested. The external surface of the cerebrum was firm. The cerebellum was soft and contained a coagulum of blood, (about 1½℥.) The substance of the cerebellum was discolored and pulpy. The lateral ventricles were filled with coagula.

Dr. White thought the case extraordinary in consideration of the existence of pregnancy and the age of the patient, (26 years.) It was a question whether the puerperal fever of last year had any agency in its development. The cerebellum had long suffered considerable organic change, yet the general health was but slightly disturbed.

The Association then adjourned.

AUSTIN FLINT, Jr., M. D.,
Secretary.

